# Rapid detection of fetal compromise using input length invariant deep learning on fetal heart rate signals

**DOI:** 10.1038/s41598-024-63108-6

**Published:** 2024-06-01

**Authors:** Lochana Mendis, Marimuthu Palaniswami, Emerson Keenan, Fiona Brownfoot

**Affiliations:** 1 Department of Electrical and Electronic Engineering, The University of Melbourne, Parkville, 3010 VIC Australia; 2 Obstetric Diagnostics and Therapeutics Group, Department of Obstetrics and Gynaecology, The University of Melbourne, Heidelberg, 3084 VIC Australia

**Keywords:** Biomedical engineering, Hypoxia, Pregnancy outcome

## Abstract

Standard clinical practice to assess fetal well-being during labour utilises monitoring of the fetal heart rate (FHR) using cardiotocography. However, visual evaluation of FHR signals can result in subjective interpretations leading to inter and intra-observer disagreement. Therefore, recent studies have proposed deep-learning-based methods to interpret FHR signals and detect fetal compromise. These methods have typically focused on evaluating fixed-length FHR segments at the conclusion of labour, leaving little time for clinicians to intervene. In this study, we propose a novel FHR evaluation method using an input length invariant deep learning model (FHR-LINet) to progressively evaluate FHR as labour progresses and achieve rapid detection of fetal compromise. Using our FHR-LINet model, we obtained approximately 25% reduction in the time taken to detect fetal compromise compared to the state-of-the-art multimodal convolutional neural network while achieving 27.5%, 45.0%, 56.5% and 65.0% mean true positive rate at 5%, 10%, 15% and 20% false positive rate respectively. A diagnostic system based on our approach could potentially enable earlier intervention for fetal compromise and improve clinical outcomes.

## Introduction

Annually, about 2 million babies are stillborn worldwide and over 40 per cent of them occur during labour^1^. However, many stillbirths are preventable with high-quality monitoring during pregnancy^2^ and timely obstetric care during labour^3^. A major cause of stillbirths is intrapartum asphyxia^4^ which occurs as a result of inadequate oxygen supply to the baby during the labour and delivery process. The fetal oxygen supply relies entirely on the uteroplacental blood flow across the umbilical cord. Any disruptions or complications in this flow can initially result in reduced oxygen concentration in fetal arterial blood and ultimately in the tissues, a condition known as hypoxia^5^. This oxygen deprivation can cause significant long-term consequences for the baby, including brain damage, developmental delays, and in severe cases, even death^6^. Therefore, when the fetus experiences compromise due to low levels of oxygen in the body, its sympathetic and parasympathetic nervous systems try to compensate by varying the cardiac output and prioritizing the blood supply to vital organs such as the heart and the brain^7,8^. Since these fetal responses are associated with changes in the fetal heart rate (FHR), it is common practice to monitor the FHR during labour to identify FHR abnormalities and prevent adverse outcomes for the baby^5^.

**Figure 1:**
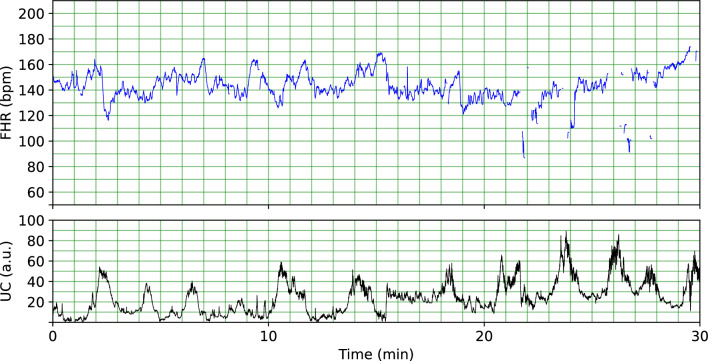
A 30 mins snippet of a cardiotocography recording showing the fetal heart rate (FHR) in blue (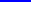) and the uterine contractions (UC) in black (—). The FHR is shown in beats per minute (bpm) and UC is shown in arbitrary units (a.u.).

Cardiotocography (CTG) is the primary technique used in obstetric care for the simultaneous monitoring of the FHR and uterine contractions (UC) continuously during pregnancy and labour. An extract of a CTG recording is shown in Fig. 1 showing the FHR and UC over a 30 mins period. Despite its widespread clinical use, CTG recordings are often visually evaluated based on clinical guidelines leading to inconsistent interpretations among clinicians (inter-observer variability) and even by the same clinician at different points in time (intra-observer variability)^9–11^. This has led to an increase in false positive rates in detecting fetal compromise which is cited as one of the main reasons for the increasing rates of caesarean section and instrumental births^12,13^. In fact, the sensitivity of detecting fetal compromise in clinical practice is quite low, typically ranging from 31 to 48% with a false positive rate of 16–21%^14,15^. This poor sensitivity in detecting fetal compromise can lead to delayed delivery for babies who are at risk. Consequently, an ongoing clinical need remains for an objective and consistent computer-based system for interpreting CTGs to ensure positive neonatal outcomes.

With the aim of addressing this clinical need, several automated fetal compromise detection methods have been proposed over the last decade^16–19^. In particular, feature-based methods such as the decelerative capacity of phase rectified signal averaging (DC_PRSA_) which quantifies the downward trend of FHR, has shown strong association for predicting fetal compromise^19^. A range of other FHR features have been proposed^20–22^, with three specific FHR features showing strong classification performance using sparse feature selection^17^ including: i) median absolute deviation of FHR from baseline (MAD_dtrd_), which quantifies the average depth of decelerations; ii) $\beta$_0_ parameter, which represents the FHR baseline, and iii) Hurst parameter (H), which quantifies variability of the FHR.

Over the last few years, deep-learning methods have also been proposed to address this clinical problem, showing promising classification performance^14,23–26^. Some of these deep-learning methods utilize both FHR and UC signals^14,24,27^ while others focus solely on analysing FHR^25,26,28^, potentially due to the lower quality of UC signals acquired using an external tocodynamometer^23^. A retrospective study by Bakker et al.^29^ found that only 2% of UC recordings captured using an external tocodynamometer were deemed adequate during the first stage of labour compared to 40% deemed adequate using an intrauterine pressure catheter. Notably, the current state-of-the-art deep-learning model for detecting fetal compromise, a multimodal convolutional neural network (MCNN)^14^, integrates both FHR and UC signals along with a quality vector. However, the study developing the MCNN acknowledges that 76% of UC signals in their private dataset were not used in analysis due to poor quality and were treated as zeros. Although both FHR and UC are evaluated in clinical settings, the role of the acquisition method and reliability of UC signals for clinical decision making remains under debate^30^. Therefore, in this work, we focus on developing new methods for solely evaluating FHR signals for the detection of fetal compromise.

Despite the advancements in computerised detection of fetal compromise, existing methods often utilize a fixed FHR segment length for analysis typically ranging from 15 to 60 min at the conclusion of labour. For example, the MCNN model provides a prediction after analysing a 60 mins FHR segment directly prior to birth. Therefore, the fetal compromise is detected at the end of labour giving less time for the clinician to intervene and take appropriate action to prevent death or irreversible injury. Further, the severity of the fetal injuries due to these compromise events worsens with the progression of time^8,31^ and therefore early identification of these events using computerised approaches is highly desired by clinicians.

In this regard, a prior study^17^ suggests that future work should evaluate the evolution of FHR features over time rather than evaluating a static segment. A system of this nature holds greater clinical significance, as it has the potential to identify fetal compromise events at an earlier stage, thereby facilitating timely clinical intervention^23^. Sisporto 4.0^32^ and OxSys 1.5^33^ are two feature-based continuous CTG analysing systems that use this concept to detect fetal compromise. Sisporto 4.0 starts the combined analysis of CTG and electrocardiogram ST event signals (ST segment of the PQRST waveform of the electrocardiogram) automatically after 10 min of acquisition and gives real-time alerts of the anomalies detected every minute. On the other hand, OxSys 1.5 analyses 15 mins windows of the CTG with 5 mins steps to trigger alarms of fetal compromise. This system is largely based on the single feature DC_PRSA_. The Sisporto system was evaluated in a randomised control trial and it concluded that no significant reduction in the incidence of metabolic acidosis was observed^34^. Similarly, a retrospective study on the OxSys 1.5 system also did not achieve significant performance difference over the clinical practice in detecting fetal compromise^33^. That is, the performance of both systems has not yet demonstrated significant improvement to justify widespread clinical application.

Therefore, in this study, we develop and benchmark an input-length invariant deep learning model architecture to determine whether fetal compromise could be detected much earlier in labour while achieving better or similar classification performance to state-of-the-art methods. To validate the performance of the proposed method we compare it against the performance of MCNN and the four identified FHR features DC_PRSA_, MAD_dtrd_, $\beta _{0}$ and H. Additionally, we evaluate the effect of signal loss in the FHR recordings on the performance of the proposed method.

## Results

### Data structure and distribution of signal loss in CTU-UHB dataset

**Figure 2:**
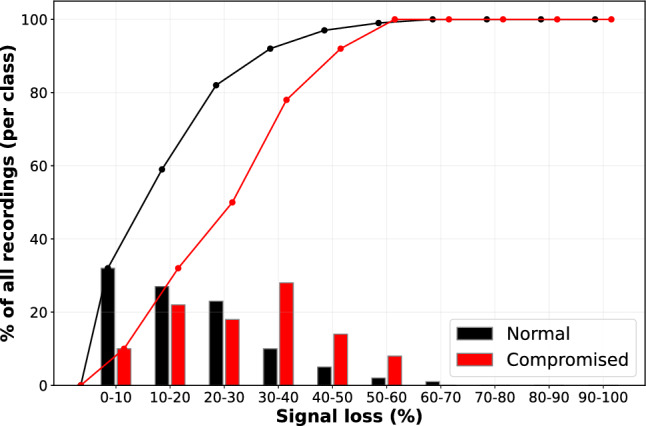
Distribution of recordings of the CTU-UHB dataset that belong to normal and compromised classes at different signal loss percentages from 10-100%. The line graph shows the cumulative distributions of the recordings according to signal loss percentage bands.

In this study, we utilized the open-access CTU-UHB intrapartum CTG dataset. It consists of 552 raw CTG recordings with UC and FHR sampled at 4Hz. We studied the latest 60 min of FHR recordings from the CTU-UHB dataset, as done in previous deep-learning studies for detecting fetal compromise^14,35^. The umbilical cord pH (pH) threshold of 7.05 was employed as the classification criterion to categorize the CTU-UHB dataset into normal and compromised classes, with pH < 7.05 indicative of compromised cases. This created a highly imbalanced class distribution where only 40 recordings belong to the compromised class. We evaluated the percentage of recordings belonging to each class at different signal loss percentages from 10 to 100%. Here, the signal loss percentage is calculated by taking the percentage of zero FHR values in an FHR recording. The signal loss distribution is shown in Fig. 2. As can be seen, 59% of the recordings in the normal class and 32% of the recordings in the compromised class have signal loss below 20%. This shows that the recordings of the compromised class contain more signal loss compared to the normal class.

### Comparison of the FHR-LINet model with the MCNN

We propose a novel input-length invariant deep learning model called FHR-LINet, comprising three parallel convolution layers followed by two consecutive convolution layers. The output from the last convolution layer undergoes global average pooling and passes through fully connected layers for classification. This architecture enables prediction on variable input length FHR segments without the need for model retraining. Alongside the FHR-LINet model, we employed three decision tree-based approaches to evaluate the FHR recording segments over time and compared their performance with the MCNN. These approaches divide a 60 mins FHR recording into multiple segments and evaluate them individually to give a single outcome to the entire FHR recording. Approach 1 evaluates individual 15 mins segments with 5 mins steps of the 60 mins FHR recording. Approach 2 evaluates cumulative segments of FHR, starting with the initial 15 min and incrementally increasing the evaluation window by 5 min at each step. Approach 3 evaluates the entire FHR recording as a single segment, which provides a direct comparison to the MCNN approach.

The MCNN achieves current state-of-the-art classification performance on the CTU-UHB dataset with true positive rate (TPR) of 33%, 48%, 58% and 65% at 5%, 10%, 15% and 20% false positive rate (FPR) respectively^14^. It was trained on the last 60 min of over 30,000 CTG recordings irrespective of labour status, taken from the proprietary Oxford CTG dataset. It uses three independent inputs: FHR, UC, and a signal quality vector to perform classification. An inherent requirement of the MCNN architecture is for the prediction length of the analysed segment to match the input length utilized during training.

We compared the performance of the proposed Approaches 1, 2 and 3 using the FHR-LINet model against the MCNN using the following performance metrics: the true positive rate at 5%, 10%, 15% and 20% false positive rate, along with a novel metric called the time to predict (TTP). TTP measures the time taken to detect fetal compromise from the beginning of FHR recording. In clinical practice, achieving a lower TTP is crucial as it allows more time for clinicians to intervene and assist at-risk fetuses. Approaches 1 and 2 measure TTP as the time elapsed from the start of FHR recording to the first window detecting fetal compromise. In contrast, Approach 3 which is identical to the MCNN approach, requires analysing a fixed segment length before providing a prediction, thereby fixing its TTP value at the input size used for training. As the original MCNN trained on the Oxford dataset utilises a fixed input size of 60 mins, its TTP value is 60 mins.

The TPR performance of FHR-LINet using the three decision tree-based approaches in comparison to the original MCNN trained on Oxford dataset and our implementation of MCNN trained on CTU-UHB dataset are shown in Table 1. The results for methods trained on CTU-UHB were obtained after evaluating their performance using 5 times repeated 5-fold cross-validation. As can be seen, our FHR-LINet model trained with augmented 30 mins data when used with Approach 1 achieved mean TPRs of 27.5%, 40.5%, 49.5% and 57.5% at 5%, 10%, 15% and 20% FPRs respectively. Using Approach 2, FHR-LINet achieved mean TPRs of 27.5%, 45%, 56.5% and 65% at 5%, 10%, 15% and 20% FPRs respectively. Lastly, FHR-LINet using Approach 3 achieved mean TPRs of 29.5%, 44%, 57.5% and 65% at 5%, 10%, 15% and 20% FPRs respectively. Notably, at 20% FPR our FHR-LINet trained with augmented 30 mins data in Approach 2 and 3 showed the same performance to MCNN trained on Oxford dataset while achieving comparable performance at 5%, 10%, and 15% FPR despite being trained on a much smaller CTU-UHB dataset compared to the Oxford dataset. FHR-LINet trained with augmented 30 mins data when employed in Approach 1 showed lower performance in terms of TPR compared to MCNN.

**Table 1:** Comparison of TPR performance at 5%, 10%, 15% and 20% FPRs for Approaches 1, 2 and 3 with our FHR-LINet method, the state-of-the-art MCNN model and FHR feature-based classification using Logistic Regression. The TPR results are given as mean ± standard deviation calculated from five times repeated 5-fold cross-validation. Reported results are calculated when no parts of the signal are removed based on signal loss and evaluated on 60 mins data unless highlighted otherwise.

	True Positive Rate (TPR) - Mean ± Standard Deviation											
	Approach 1				Approach 2				Approach 3			
	5%FPR	10%FPR	15%FPR	20%FPR	5%FPR	10%FPR	15%FPR	20%FPR	5%FPR	10%FPR	15%FPR	20%FPR
Our Work												
FHR-LINet (trained on CTU-UHB,augmented 30 min)	27.5 ± 4.7	**40.5** ± **5.4**	**49.5** ± **4.5**	**57.5** ± **6.6**	**27.5** ± **9.5**	**45.0** ± **8.7**	**56.5** ± **4.5**	**65.0** ± **8.3**	29.5 ± 8.9	44.0 ± 6.3	57.5 ± 7.3	**65.0** ± **10.3**
FHR-LINet(trained on CTU-UHB, 60 min)	5.0 ± 4.0	31.0 ± 3.8	41.5 ± 2.2	48.5 ± 2.9	24.0 ± 4.2	42.0 ± 5.7	52.0 ± 2.7	58.5 ± 3.8	24.0 ± 4.2	42.0 ± 5.7	52.0 ± 2.7	58.5 ± 3.8
FHR-LINet(trained on CTU-UHB, first 45 min)	14.5 ± 5.1	26.0 ± 6.3	33.0 ± 5.1	42.0 ± 2.7	24.5 ± 6.0	38.0 ± 3.3	49.0 ± 1.4	57.5 ± 3.5	21.0 ± 5.2^e^	28.0 ± 6.0^e^	39.5 ± 3.3^e^	51.5 ± 2.9^e^
FHR-LINet (trained on CTU-UHB, first 30 min)	10.0 ± 4.0	21.5 ± 5.8	32.5 ± 7.9	40.0 ± 3.1	19.0 ± 9.6	28.0 ± 6.5	41.5 ± 4.9	50.5 ± 4.8	15.0 ± 6.8^f^	23.0 ± 5.7^f^	28.5 ± 3.8^f^	36.0 ± 4.2^f^
MCNN												
MCNN^a^(trained on Oxford,60 min)	–	–	–	–	-	–	–	–	**33**^**c**^	**48**^**c**^	**58**^**c**^	**65**^**c**^
MCNN^b^(trained on CTU-UHB, 60 min)	–	–	–	–	–	–	–	–	25.5 ± 3.3	33.0 ± 3.3	45.5 ± 3.3	51.5 ± 4.9
MCNN^b^(trained on CTU-UHB, first 45 min)	–	–	–	–	–	–	–	–	15.5 ± 10.8^e^	26.5 ± 9.9^e^	34.5 ± 9.7^e^	40.5 ± 7.6^e^
MCNN^b^(trained on CTU-UHB, first 30 min)	–	–	–	–	–	–	–	–	13.0 ± 7.2^f^	21.5 ± 8.9^f^	27.5 ± 7.1^f^	31.5 ± 6.5^f^
Feature Based Logistic Regression												
DC_PRSA_^d^	**28.0** ± **3.3**	34.0 ± 2.2	45.0 ± 2.5	53.5 ± 2.2	20.5 ± 3.3	36.5 ± 3.4	43.5 ± 2.2	47.5 ± 2.5	20.5 ± 3.3	36.5 ± 3.4	43.5 ± 2.2	47.5 ± 2.5
MAD_dtrd_^d^	3.5 ± 2.2	10.5 ± 2.7	18.5 ± 2.2	23.0 ± 2.7	7.0 ± 1.1	9.5 ± 1.1	12.5 ± 1.8	23.0 ± 1.1	7.0 ± 1.1	9.5 ± 1.1	12.5 ± 1.8	23.0 ± 1.1
$\beta$_0_^d^	16.5 ± 2.9	29.0 ± 4.5	39.5 ± 2.1	49.5 ± 2.7	11.5 ± 1.4	22.5 ± 2.5	32.0 ± 2.1	41.5 ± 3.8	11.5 ± 1.4	22.5 ± 2.5	32.0 ± 2.1	41.5 ± 3.8
H^d^	3.5 ± 1.4	7.5 ± 1.8	9.0 ± 1.4	10.0 ± 1.8	5.5 ± 3.3	7.0 ± 3.3	11.5 ± 2.9	17.5 ± 4.0	5.5 ± 3.3	7.5 ± 4.0	11.5 ± 2.9	17.5 ± 4.0
All Features^d^	26.5 ± 2.9	36.5 ± 3.8	45.0 ± 2.5	52.5 ± 3.5	26.0 ± 4.9	36.0 ± 5.2	41.0 ± 1.4	46.0 ± 1.4	26.5 ± 5.2	36.5 ± 5.2	41.0 ± 1.4	46.0 ± 1.4

a = Petrozziello et al MCNN^14^, b = Our implementation of MCNN, c = Mean and standard deviation not available, d = Trained on features calculated on 60 min window of CTU-UHB, e = Tested on first 45 mins data, f = Tested on first 30 mins data.

The best performance in each column is in bold.

The main benefit achieved using Approach 1 and Approach 2 is the lower TTP. As can be seen from Table 2, for Approach 1, the mean TTP of FHR-LINet trained with augmented 30 mins data at 5%, 10%, 15% and 20% FPR was 43, 42, 41 and 41 mins. For Approach 2, the mean TTP of FHR-LINet trained with augmented 30 mins data at 5%, 10%, 15% and 20% FPR was 45, 46, 45 and 45 mins. That is, Approach 1 and Approach 2 using FHR-LINet trained on augmented 30 mins data achieved approximately 30% and 25% reduction in the time taken to predict fetal compromise respectively. This is an improvement in TTP for both approaches compared to the 60 mins taken by MCNN trained on Oxford data, with comparable TPR for Approach 2 and lower TPR for Approach 1.

**Table 2:** Comparison of TTP performance at 5%, 10%, 15% and 20% FPRs for Approaches 1, 2 and 3 with our FHR-LINet method, the state-of-the-art MCNN model and FHR feature-based classification using Logistic Regression. The TTP results are given as mean ± standard deviation calculated from five times repeated 5-fold cross-validation. Reported results are calculated when no parts of the signal are removed based on signal loss and evaluated on 60 mins data unless highlighted otherwise.

	Time to Predict (TTP) - Mean ± Standard Deviation											
	Approach 1				Approach 2				Approach 3			
	5%FPR	10%FPR	15%FPR	20%FPR	5%FPR	10%FPR	15%FPR	20%FPR	5%FPR	10%FPR	15%FPR	20%FPR
Our work												
FHR-LINet (trained on CTU-UHB, augmented 30 min)	43.0 ± 2.0	42.0 ± 1.0	41.0 ± 2.0	41.0 ± 3.0	**45.0** ± **3.0**	**46.0** ± **3.0**	**45.0** ± **2.0**	**45.0** ± **1.0**	60.0 ± 0.0	60.0 ± 0.0	60.0 ± 0.0	60.0 ± 0.0
FHR-LINet (trained on CTU-UHB, 60 min)	44.0 ± 3.0	47.0 ± 2.0	45.0 ± 1.0	43.0 ± 1.0	56.0 ± 0.0	55.0 ± 1.0	55.0 ± 1.0	54.0 ± 1.0	60.0 ± 0.0	60.0 ± 0.0	60.0 ± 0.0	60.0 ± 0.0
FHR-LINet(trained on CTU-UHB, first 45 min)	48.0 ± 3.0	49.0 ± 2.0	48.0 ± 1.0	46.0 ± 0.0	57.0 ± 0.0	57.0 ± 0.0	56.0 ± 1.0	56.0 ± 1.0	45.0 ± 0.0^e^	45.0 ± 0.0^e^	45.0 ± 0.0^e^	45.0 ± 0.0^e^
FHR-LINet (trained on CTU-UHB, first 30 min)	42.0 ± 3.0	43.0 ± 2.0	41.0 ± 3.0	40.0 ± 2.0	57.0 ± 1.0	57.0 ± 1.0	56.0 ± 1.0	56.0 ± 0.0	**30.0** ± **0.0**^**f**^	**30.0** ± **0.0**^**f**^	**30.0** ± **0.0**^**f**^	**30.0** ± **0.0**^**f**^
MCNN												
MCNN^a^ (trained on Oxford, 60 min)	–	–	–	–	–	–	–	–	60^c^	60^c^	60^c^	60^c^
MCNN^b^ (trained on CTU-UHB, 60 min)	–	–	–	–	–	–	–	–	60.0 ± 0.0	60.0 ± 0.0	60.0 ± 0.0	60.0 ± 0.0
MCNN^b^ (trained on CTU-UHB, first 45 min)	–	–	–	–	–	–	–	–	45.0 ± 0.0^e^	45.0 ± 0.0^e^	45.0 ± 0.0^e^	45.0 ± 0.0^e^
MCNN^b^ (trained on CTU-UHB, first 30 min)	–	–	–	–	–	–	–	–	**30.0** ± **0.0**^f^	**30.0** ± **0.0**^f^	**30.0** ± **0.0**^f^	**30.0** ± **0.0**^f^
Feature Based Logistic Regression												
DC_PRSA_^d^	49.0 ± 1.0	47.0 ± 1.0	47.0 ± 1.0	46.0 ± 0.0	53.0 ± 1.0	55.0 ± 0.0	56.0 ± 0.0	56.0 ± 0.0	60.0 ± 0.0	60.0 ± 0.0	60.0 ± 0.0	60.0 ± 0.0
MAD_dtrd_^d^	49.0 ± 1.0	46.0 ± 1.0	43.0 ± 1.0	41.0 ± 0.0	58.0 ± 0.0	58.0 ± 0.0	57.0 ± 0.0	57.0 ± 0.0	60.0 ± 0.0	60.0 ± 0.0	60.0 ± 0.0	60.0 ± 0.0
$\beta$_0_^d^	**28.0** ± **2.0**	**27.0** ± **2.0**	**28.0** ± **1.0**	**28.0** ± **0.0**	59.0 ± 0.0	59.0 ± 0.0	59.0 ± 0.0	59.0 ± 0.0	60.0 ± 0.0	60.0 ± 0.0	60.0 ± 0.0	60.0 ± 0.0
H^d^	38.0 ± 1.0	37.0 ± 1.0	35.0 ± 1.0	35.0 ± 0.0	60.0 ± 0.0	60.0 ± 0.0	60.0 ± 0.0	60.0 ± 0.0	60.0 ± 0.0	60.0 ± 0.0	60.0 ± 0.0	60.0 ± 0.0
All features^d^	49.0 ± 1.0	48.0 ± 0.0	47.0 ± 1.0	46.0 ± 0.0	53.0 ± 1.0	54.0 ± 0.0	54.0 ± 1.0	55.0 ± 0.0	60.0 ± 0.0	60.0 ± 0.0	60.0 ± 0.0	60.0 ± 0.0

a = Petrozziello et al MCNN^14^, b = Our implementation of MCNN, c = Mean and standard deviation not available, d = Trained on features calculated on 60 min window of CTU-UHB, e = Tested on first 45 mins data, f = Tested on first 30 mins data.

The best performance in each column is in bold.

### Comparison of the FHR-LINet model and MCNN trained on different input lengths

To compare the performance of MCNN at different fixed segment lengths and thus provide a comparison for similar TTP, we implemented and trained the MCNN on the CTU-UHB using three variations: the entire 60 mins segment length, as well as the first 45 mins only, and the first 30 mins only of the entire 60 mins segment. This setup yielded corresponding TTP values of 60, 45, and 30 mins respectively. Similarly, we trained the FHR-LINet model in these same three variations in addition to using augmented 30 mins data as presented in the previous section.

The comparison of our implementation of MCNN when trained on the public CTU-UHB dataset was restricted to Approach 3. As can be seen from Table 1, our implementation of MCNN trained on 60 mins segments using Approach 3, achieved mean TPRs of 25.5%, 33%, 45.5% and 51.5% at 5%, 10%, 15% and 20% FPRs respectively. This performance is lower compared to the reported performance of the MCNN trained on Oxford data. We note that the CTU-UHB dataset is approximately 50 times smaller compared to the Oxford dataset used for training the original MCNN. Furthermore, the mean TPR performance of MCNN showed a decreasing trend when we reduced the training segment length from 60 mins to the first 45 and first 30 mins. Similarly, FHR-LINet demonstrated a similar trend but with improved TPR performance when trained and tested with shorter fixed segment lengths in Approach 3. Conversely, FHR-LINet showed superior performance across all 3 approaches when trained with augmented 30 mins data.

Moreover, we noted that the FHR-LINet, when trained on 60 mins, the first 45 mins, and the first 30 mins window segments, showed a TTP of approximately 56 mins using Approach 2, compared to TTP of approximately 45 mins taken by FHR-LINet trained on augmented 30 mins data using Approach 2. This suggests that training the FHR-LINet with a larger number of samples by utilizing augmented 30 mins enhances both TPR and TTP performance compared to training with a smaller training dataset.

### Comparison of the FHR-LINet model with FHR features

We compared the TPR and TTP performances of the proposed FHR-LINet model with four FHR features (DC_PRSA_, MAD_dtrd_, $\beta$_0_ and H) individually, as well as with all four features combined using a Logistic Regression (LR) classifier. As can be seen in Table 1, our FHR-LINet trained on augmented 30 mins outperformed feature-based LR in all three Approaches with only one exception at 5% FPR in Approach 1. It can be seen that the DC_PRSA_ and $\beta$_0_ features exhibited stronger TPR performance compared to other FHR features in all three approaches, with DC_PRSA_ being the top performer. With Approach 1, DC_PRSA_ achieved mean TPRs of 28%, 34%, 45% and 53.5% at 5%, 10%, 15% and 20% FPR respectively.

In terms of TTP performance, as demonstrated in Table 2, our FHR-LINet model trained on augmented 30 mins data in Approach 2 outperformed the feature-based LR models. However, we observed that the TTP performance of $\beta$_0_ feature in Approach 1 showed notable improvement in comparison to all other methods with reasonable TPR. It achieved mean TTP values of 28, 27, 28 and 28 mins at 5%, 10%, 15% and 20% FPR respectively. Furthermore, in the FHR feature-based analysis using LR, we observed that Approach 1 demonstrated superior TTP performance compared to Approaches 2 and 3.

### Breakdown of TTP into true positives and false positives

**Figure 3:**
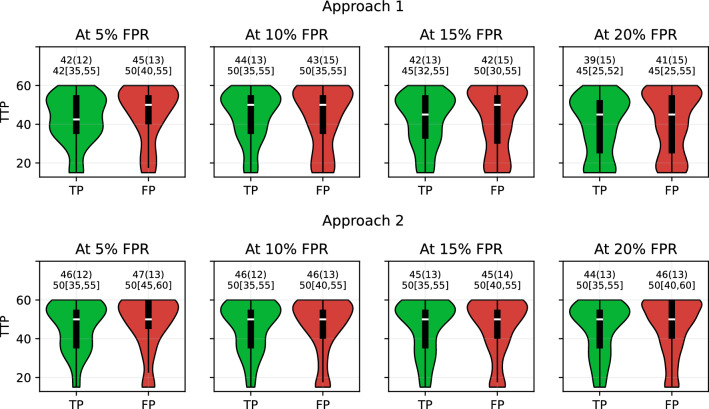
Distribution of TTP values of True positives (TP) and False positives (FP) using violin plot for Approach 1 and Approach 2 at 5%, 10%, 15% and 20% FPR using FHR-LINet with augmented 30 mins training data. The TTP values are given in mean (standard deviation) and median [first quartile, third quartile] above each violin plot.

The TTP values were computed by considering all positive predictions, encompassing both true and false positives. To illustrate the relative contributions of true positives and false positives towards the overall TTP, we plotted the distribution of TTP predictions of FHR-LINet trained with augmented 30 mins using violin plots, as shown in Fig. 3. In the figure, we show the mean and standard deviation of TTP, as well as the median and interquartile range. As can be seen, in Approach 1, true positives exhibit a lower or equal median TTP compared to false positives. In Approach 2, while the medians are identical for true positives and false positives, the first quartile of true positives is lower than that of false positives.

### Comparison of Approaches 1, 2, and 3 using McNemar’s test

McNemar’s test^36^ is a hypothesis test that allows for the comparison of two classification methods by focusing on differences in class predictions. Using a 2x2 contingency table, we tabulated the number of correctly and incorrectly classified compromised cases for different Approaches. By calculating the p-value using McNemar’s test, we compared the TPRs achieved by our proposed FHR-LINet model trained on augmented 30 mins data between Approaches 1, 2 and 3 at 5%, 10%, 15%, and 20% FPR thresholds.

The results of McNemar’s test indicated that the TPRs at 5%, 10%, 15% and 20% FPR were not statistically significant (p $> 0.05$) when comparing Approach 2 and Approach 3. However, the TPR at 15% FPR when comparing Approach 1 and Approach 2, and the TPRs at 15% and 20% FPR when comparing Approach 1 and Approach 3, were statistically significant (p $< 0.05$) while the remaining comparisons were not statistically significant. Therefore, the classification performance obtained with the three approaches using the FHR-LINet model would need to be re-evaluated in a future cohort with a larger sample size to conclusively determine the superior approach in terms of TPR.

### Comparison of Approaches 1, 2 and 3 at different signal loss thresholds

To examine the impact of FHR signal loss on the performance of our methods, we conducted an evaluation of the TPR at various signal loss percentages, ranging from 10 to 100%. During the training process with augmented 30 mins data, only the FHR segments that met the selected signal loss threshold were retained in the training data. In contrast, the FHR window segments in the FHR recordings of the testing data that did not meet the signal loss threshold were classified as normal.

**Figure 4:**
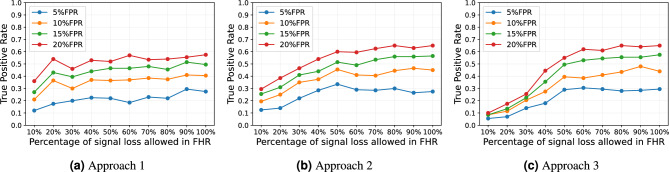
The variation of the true positive rate at 5%, 10%, 15% and 20% false positive rate for Approach 1, 2 and 3 at different percentages of signal loss allowed in FHR using FHR-LINet with augmented 30 mins training data.

As illustrated in Fig. 4, all three approaches exhibit an upward trend in TPR as the allowance for signal loss in the FHR recording increases. However, Approaches 2 and 3 showed a steeper increment from 10 to 50% signal loss allowed compared to Approach 1. When a 10% signal loss was permitted, Approach 1 yielded its lowest TPRs of 12% at 5% FPR, 21% at 10% FPR, 27% at 15% FPR, and 36% at 20% FPR. Similarly, at the same signal loss threshold, both Approaches 2 and 3 also yielded their lowest TPRs of 12.5%, 19.5%, 25.5%, 29.5% and 5.5%, 8.5%, 8.5%, 10% respectively at 5%, 10%, 15% and 20% FPR. In all three approaches, the majority of the highest TPR performance at 5%, 10%, 15% and 20% FPRs were observed when 100% signal loss was permitted. The TPRs corresponding to 100% signal loss threshold are given in Table 1.

**Figure 5:**
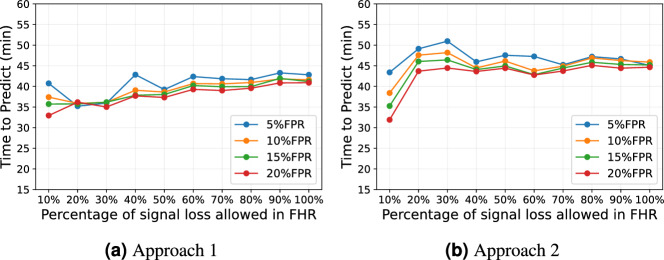
The variation of TTP at 5%, 10%, 15% and 20% false positive rate for Approach 1 and 2 at different percentages of signal loss allowed in FHR using FHR-LINet with augmented 30 mins training data.

As shown in Fig. 5, the TTP in Approach 1 increases as more signal loss is allowed in the FHR. In contrast, Approach 2 shows an increase in TTP when the signal loss threshold increases from 10 to 30%, but then drops to around 45 mins and remains relatively stable up to 100% signal loss allowed. The TTP for Approach 3 is constant at 60 mins. When 10% signal loss was allowed, Approach 2 achieved its lowest TTPs of 43, 38, 35, and 32 mins at FPRs of 5%, 10%, 15% and 20% respectively. However, Approach 1 achieved its lowest TTPs of 35, 36, and 35 mins at FPRs of 5%, 10%, and 15% when 20% signal loss was allowed, and a TTP of 33 mins at FPR of 20% when 10% signal loss was allowed.

## Discussion

In this study, we developed an input-length invariant deep learning model called FHR-LINet, together with three decision tree-based evaluation approaches to rapidly detect fetal compromise in FHR recordings. We assessed the performance of our FHR-LINet model using a fetal compromise threshold of pH $< 7.05$ on the CTU-UHB dataset and compared its performance with a state-of-the-art MCNN deep learning model^35^, and four FHR features (DC_PRSA_, MAD_dtrd_, $\beta$_0_, and H)^17,19^. We demonstrated that our FHR-LINet model achieved comparable classification performance to the MCNN using a decision tree approach which evaluates cumulative FHR segments (Approach 2), achieving approximately 25% reduction in the time to predict (TTP) fetal compromise. A decision tree approach which evaluates individual 15 mins FHR segments in 5 mins steps (Approach 1), achieved lower classification performance compared to Approach 2 while achieving a 30% reduction in TTP. The classification performance of the FHR feature-based methods was lower than both the FHR-LINet and MCNN methods, except for the performance of DC_PRSA_ at a 5% FPR.

**Figure 6:**
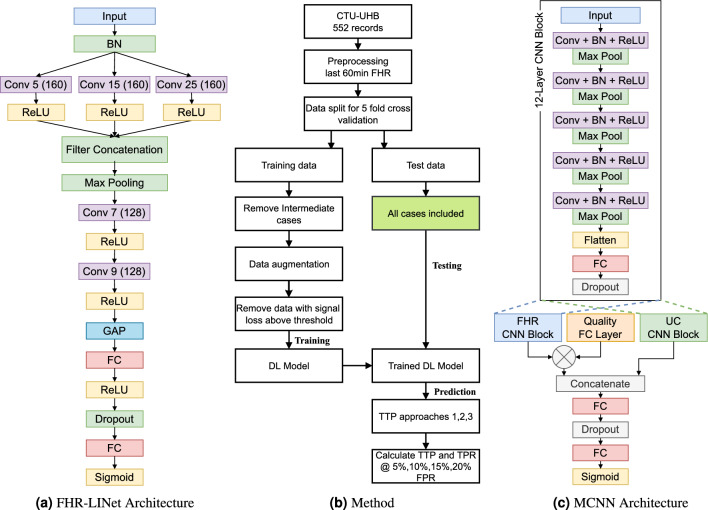
(**a**) Overview of the novel input length invariant deep learning model architecture. One-dimensional FHR recordings of any length can be used as input to this model to detect the probability of fetal compromise. GAP and Conv i (j) refer to the global average pooling layer and convolutional layers with kernel size i and the number of filters j, respectively. (**b**) Overview of the method used to train and evaluate the proposed deep learning model. (**c**) Overview of the MCNN architecture used in this study.

As shown in Fig. 6, our proposed deep-learning model FHR-LINet is based on a CNN architecture as it has previously proven its superiority in fetal compromise detection compared to other architectures^14,24,25,35^. Petrozziello et al.^35^ particularly evaluated long-short-term memory networks (LSTM) and convolutional neural networks (CNN) and showed that CNNs have better classification performance in detecting fetal compromise than LSTMs^15,33^. Our FHR-LINet model uses three parallel branches of convolution layers with different dilated kernel sizes taking inspiration from the inception model to capture multiscale information from FHR signals^37,38^. The convolutional layers in CNNs are by default invariant to the input length, as the trainable kernel weights do not depend on the input size of the layer. However, typical CNNs have a flattening layer followed by several fully connected layers with fixed input sizes that restrict them to a specific input length. Therefore, the inclusion of the global average pooling (GAP) layer in our model removes this restriction as it averages the temporal dimension of the FHR features found from the convolution regardless of the input size^39^. The novelty of integrating the inception module and GAP layer into the CNN makes our model invariant to input length and can capture multiscale information of FHR at the same time. A specific advantage of this architecture is that our model can be trained and tested on different input lengths, allowing for augmenting the training data into multiple segments of variable lengths to increase the number of training samples.

Our proposed model in Approach 1 evaluates FHR recordings over time by evaluating 15 mins window segments with a stride of 5 mins, whereas Approach 2 evaluates growing FHR window segments. Although these two approaches achieved good classification performance, we were not able to surpass the classification performance of MCNN trained on the Oxford dataset. However, Approach 1 and Approach 2 managed to achieve a 30% and 25% reduction in the time taken to detect fetal compromise. This is a noteworthy reduction in a clinical context as prolonged compromise events may be fatal for the baby and timely intervention is important. According to a recent retrospective cohort study^40^ and insights from animal models^41^, clinicians would typically have a window of around 5 to 30 mins before babies become extremely sick depending on the severity of the fetal compromise. Therefore, without knowledge of the type of underlying compromise, intervention as soon as 5 mins may be required highlighting the value of our approach. On this point, we note that the TTP performance of the FHR feature $\beta$_0_ was particularly strong when used with Approach 1 compared to all other methods. Therefore, different features and approaches may be used to optimise the balance between TTP and TPR/FPR and this could be explored in future work. On another note, $\beta$_0_ showed the best TPR performance compared to MAD_dtrd_ and H features although, MAD_dtrd_ was observed to be superior in prior studies^17,42^. This difference in feature performance may be attributed to variations in exclusion criteria between this study and previous ones, such as differences in FHR segment selection and the exclusion of records with signal loss which was not applied in our study.

Furthermore, we investigated how signal loss affects the performance of our proposed method. As shown in Fig. 4, the TPRs at 5%, 10%, 15% and 20% FPR show an increasing trend with the increase of the percentage of signal loss allowed in the FHR. This outcome is expected in Approach 3, as we override the prediction of FHR recordings that fail to meet the signal loss threshold to be assigned a normal label. Given that a significant number of compromised cases exhibit higher signal loss, as illustrated in Fig. 2, this may contribute to increased misclassification at lower signal loss thresholds. Nevertheless, for Approaches 1 and 2, this might not necessarily be the scenario, as the final prediction for the FHR recording depends on whether other FHR sub-segments present in the FHR recording meet the signal loss threshold. It can be seen from Fig. 5 that in Approach 1, the TTP shows an increasing trend with more signal loss allowed. This implies that a longer duration of the FHR is needed to detect fetal compromise when more signal loss is present in the FHR. However, the TTP of Approach 2 remains relatively stable at around 45 mins when signal loss greater than 30% is allowed in FHR. This could imply that the evaluation of longer FHR segments might be less affected by signal losses, as we are evaluating cumulative window segments where the FHR segments are longer in Approach 2 compared to Approach 1. However, the impact of signal loss should be further assessed in larger cohorts to comprehensively understand its implications on fetal compromise detection.

While our study presents valuable insights, it is important to acknowledge its limitations. The FHR-LINet was trained on augmented fixed window segments of 30 mins for evaluating all approaches, although our model has the potential to be trained on variable lengths. The 30 mins segments for training all approaches were selected as a middle ground to keep the training phase the same for all approaches such that effective comparisons can be made. However, an alternate approach for training our model would be to use augmented 15 mins window segments in Approach 1, augmented variable window lengths in Approach 2 and 60 mins windows for Approach 3. Finding the optimal training strategy for each approach is beyond the scope of this work and should be investigated in future work. Another crucial aspect to note is that while TTP holds clinical significance as a metric, understanding how a deep learning model achieves this lower TTP currently remains a black box for clinicians. In this regard, recent advancements in explainable artificial intelligence offer the potential to partially address this issue by enabling clinicians to correlate indicative features for classification decisions with their medical background knowledge. Particularly, the use of the GAP layer in our model would enable the future creation of class activation maps^43,44^ for the FHR recording to visualize features associated with fetal compromise and see if they align with current medical knowledge.

It is also important to note the hyper-parameters for our FHR-LINet model were chosen using empirical testing from values identified in our preliminary work^28^, the works of Petrozziello et al.^14^ and Wang et al.^44^. Finding the optimal set of hyper-parameters could be investigated in future to enhance the model performance. Regarding the construction of the decision tree for Approach 1 and Approach 2, currently we have assigned equal weighting to all segments of the FHR recording in making a decision. This approach could be problematic because fetal compromise may occur at a specific point, and before that, the FHR might be normal, or a compromised FHR might revert to normal later due to intervention from medical personnel. Therefore, finding an optimum way to weight these FHR segments should be studied in future. Other recent works^24,25^ have also employed different criteria for defining compromised cases such as pH $< 7.2$ and 1 min APGAR $< 7$^24^, and pH $< 7.15$^26^, utilized various lengths of signal for analysis (ranges from 15 to 60 mins) and showed variations in dataset selection, e.g. performance given on subset of CTU-UHB^24^. This makes comparison across existing methods problematic. This challenge of not having a standard evaluation workflow for effective comparison of fetal compromise detection methods has been studied in a recent review^23^.

Finally, according to the guidelines provided by the International Federation of Gynaecology and Obstetrics (FIGO), the CTG evaluations are typically acceptable only when the signal loss is below 20%^45^. At this signal loss threshold, we would only have 59% of the normal cases and 32% of the compromised cases in the CTU-UHB containing sufficient data for analysis. This amount of signal loss may restrict the training of deep neural networks to achieve the performance needed for widespread clinical use. Therefore, research on new technologies in CTG extraction should be investigated be to obtain good-quality recordings for FHR analysis. The non-invasive fetal electrocardiogram is one such alternative mode of FHR extraction that is showing promising results in terms of accuracy compared to invasive methods^46,47^.

In conclusion, to our knowledge, our study is the first to evaluate FHR recordings over time in detecting fetal compromise using a deep-learning model. We have introduced a novel input-length invariant deep learning model coupled with a decision tree-based evaluation approach to detect fetal compromise in evolving FHR recordings. Additionally, we have introduced a clinically relevant performance metric called time to predict to assess the effectiveness of different detection methods. By employing our approach, we have demonstrated the ability to rapidly detect fetal compromise while maintaining comparable classification performance to state-of-the-art techniques. This achievement highlights the potential of our method to improve clinical outcomes. Future research efforts should focus on exploring the practical implementation of this approach in clinical settings, with the aim of enhancing patient outcomes.

## Materials and Methods

### Dataset

The CTG dataset used in this study is the publicly available CTU-UHB Intrapartum Cardiotocography Database^48^. The CTG recordings of this dataset were collected from the obstetric ward of the University Hospital in Brno, Czech Republic between 2010 and 2012. The dataset collection was approved by the Institutional Review Board of University Hospital Brno and all participants signed informed consent. It consists of 552 CTG recordings with FHR and UC values sampled at 4 Hz. The maximum duration of these CTGs is 90 min and ends not earlier than 30 min before delivery. Most studies have used the umbilical artery cord pH as an objective measure to classify the CTG recordings into normal and compromised classes^25,42,49^.

In this study, we use only the last 60 mins FHR recordings in the CTU UHB dataset for analysis. Following the works of Petroziello et al.^14^, we selected the pH threshold of 7.05 as the criteria to divide the dataset into two classes; Normal and Compromised. That is, we classify the FHR recordings with pH $< 7.05$ as the compromised class while the recordings with pH $\ge$ 7.05 are defined as the normal class. Additionally, we define a sub-category called the intermediate class with recordings having pH $\ge$ 7.05 and pH $< 7.15$. The resulting dataset consists of only 40 recordings in the compromised class reflecting the imbalanced nature of compromised cases compared to normal cases in clinical reality.

### FHR pre-processing

In clinical practice, the fetal heart rate is monitored during labour by external monitoring through a Doppler ultrasound or directly by a fetal scalp electrode (FSE), or sometimes a combination of both methods. Demonstrating the clinical reality, the CTU-UHB dataset is also composed of FHR recordings obtained through both methods with the majority of them acquired from the Doppler ultrasound method^48^. The signals acquired from both methods are susceptible to contamination from various noise sources. The noises that arise from fetal and maternal movements, as well as displacement of the transducers, contribute to noise in signals acquired using Doppler ultrasound. On the other hand, vaginal examinations and maternal pushing contribute to the noise in the signals acquired from FSE. The intermittent measuring of the maternal heart rate also compromises the quality of the FHR in both techniques^45^. Therefore, the raw FHR data needs to be pre-processed before being analysed with computer-based deep learning methods, as low-quality data prevents stability and convergence in the learning process^50^.

In this study, we follow the pre-processing steps such as artefact removal, signal interpolation, downsampling and segment selection adopted by previous works^14,25^. The raw CTG recordings are first screened for outliers by searching for FHR values greater than 200 beats per minute (bpm) and less than 50 bpm. Next, these values are replaced by zeros. Then non-physiological values where the difference between two consecutive FHR values is greater than 25 bpm are also replaced by zeros. We define the periods of consecutive zero FHR values as gaps. The resulting FHR signal is linearly interpolated to fill only the gaps that are less than 15 s. The gaps greater than 15 s are kept unchanged as zeros. Considering that the average fetal heart beats at a maximum of 180 bpm (3 beats per second), the original sampling rate of 4 Hz may be greater than required. Therefore, to reduce the computational complexity in processing, we downsampled (averaged down) the FHR recordings to 0.25 Hz in line with the method of Petrozziello et al^14^. Finally, we selected the last 60 mins of the processed CTG recordings for the analysis such that a fair comparison can be made with the MCNN^14^.

### Input length invariant convolutional neural network architecture (FHR-LINet)

We propose an input length invariant convolutional neural network called FHR-LINet that takes the FHR signal as input and outputs the probability of compromise. An overview of the model architecture is shown in Fig. 6. The input layer is followed by a batch normalization layer to normalize the inputs by subtracting the batch mean and dividing by the batch standard deviation. It helps in speeding up the training process by reducing internal covariate shifts and providing regularization to prevent overfitting. Its output is then fed to three branches of one-dimensional convolutional layers with kernel sizes 5, 15, and 25. Each of these convolution layers has 160 filters with the rectified linear unit (ReLU) as their activation function. This allows capturing multiscale information from the input FHR signals^38^. The outputs of the convolutional layers are then concatenated in the filter dimension and passed through a one-dimensional maximum pooling layer with (pool_size = 2) to downsample the temporal data. This is then followed by two one-dimensional convolutional layers having 128 filters with filter sizes 7 and 9 respectively. Next, the temporal average of the features of each unit in the last convolutional layer is taken by a global average pooling layer^39^. A fully connected layer with 64 nodes is used as the next layer followed by a dropout layer. The final layer consists of a single neuron with a sigmoid activation function. The sigmoid transformation is chosen in the last layer because fetal compromise detection is formulated as a binary classification problem.

### Training the FHR-LINet model

The deep learning models were constructed utilizing the Tensorflow version 2 framework and underwent training for a total of 65 epochs, employing a mini-batch size of 32. To tackle class imbalance in the CTU-UHB dataset, class weights are added to the loss function to give more weightage to the losses that occur from the incorrect classification of the minority class. In training the model, we adopted the binary cross entropy error as the loss function and Adam as the optimizer function with a learning rate of 0.0001. The 5-fold stratified cross-validation is used as the training method. This divides the dataset into five folds and at one time, four of them are used as the training folds while the remaining fold is used as the testing fold. Five groups of such train and test folds are formed such that each individual fold becomes the testing fold at least once.

The intermediate cases are a heterogeneous and challenging group to detect where the occurrence of compromise events is not well established^14,51^. Therefore, intermediate cases in the training data are removed in training the model so that possible errors in the labels of the training data are minimized. However, no such data removal is done to the testing data. To increase the number of data samples in the training data, we augmented the training data by creating seven overlapping 30 mins windows with a stride of 5 mins for each 60 mins training data and assigned them the same label as the parent 60 mins FHR signal. An overview of the method used for the training and evaluation of the proposed deep learning model is shown in Fig. 6.

### Evaluation of the performance of the FHR-LINet model with three novel Approaches

To compare the performance of our proposed model against the current state-of-the-art performance achieved by the MCNN, we use three approaches as mentioned below. Additionally, we introduce a novel performance metric that has clinical relevance called time to predict (TTP) in evaluating the performance of these approaches for fetal compromise detection.

**Figure 7:**
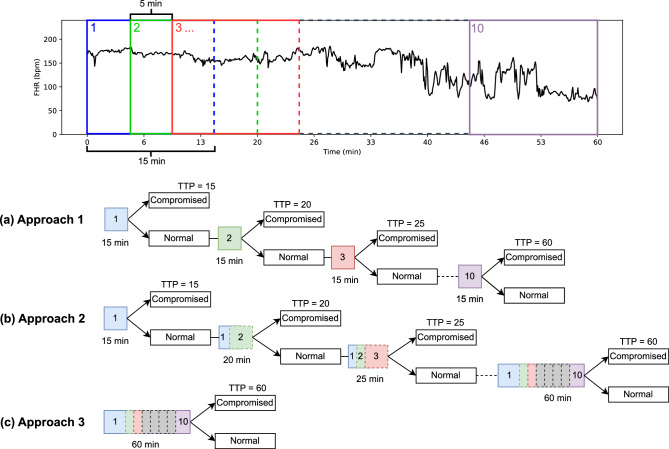
An overview of the TTP approaches in evaluating a 60 mins FHR recording. (**a**) Approach 1: Evaluating 15 mins sliding windows with a stride of 5 mins, (**b**) Approach 2: Evaluating cumulative windows starting from 15 mins and growing by 5 mins, (c) Approach 3: Evaluating the entire 60 mins signal.

- **Approach 1** - Evaluation is performed on 15 mins sliding windows with a 5 mins step over, starting from the beginning of signal for each FHR recording of the testing data as shown in Fig. 7. If any of the windows of a recording is predicted as positive, the complete FHR recording is classified as positive; otherwise, it is classified as normal. The TTP value of the recording is the time duration from the start of the FHR recording to the first positively predicted sliding window segment.
- **Approach 2** - Evaluation is performed on cumulative windows that increase in length by 5 mins increments, starting from a 15 mins window of the signal for each FHR recording in the testing data. The TTP is calculated similarly to Approach 1.
- **Approach 3** - Evaluation is performed on the entire evaluating length of the recording which is a direct comparison to MCNN. Here the TTP value is fixed at the entire length of the evaluation.

Each approach undergoes three main steps to provide the final performance metrics. The predictions of a binary classification depend on the threshold value which acts as the decision boundary for the predicted probabilities. Therefore, as the first step, the threshold values that give 5%, 10%, 15% and 20% FPRs are found using the prediction probabilities obtained from each test data fold. Next, these thresholds are used as the decision boundary to classify the prediction probabilities of the test data folds into normal and compromised classes. The TTP values for the test data folds are calculated as defined in each of the approaches. Finally, the TPRs for 5%, 10%, 15% and 20% FPRs are calculated using the class predictions of all test folds such that we get a single TPR value for each 5%, 10%, 15% and 20% FPR. A single TTP measure for each FPR is calculated across all test folds by taking the average TTP values of FHR recordings that are predicted positive including the false positives.

When searching the threshold values for Approaches 1 and 3, we used a step size of 0.001 and iteratively reduced the threshold from 1.0 until we reached the desired FPRs. However, using a single threshold value for each desired FPR in Approach 2 was inappropriate as it evaluates windows of variable lengths. Consequently, we evaluated the variation of the threshold values for 5%, 10%, 15% and 20% FPRs at different window lengths ranging from 15 to 60 mins using Approach 1. We identified that these threshold values decrease as the window length increases. To ensure the robustness of our evaluation, we performed five times repeated 5-fold cross-validation on Approach 1, calculating the average threshold values for the specified FPRs at different window lengths. Then, using these values in linear regression, we obtained the approximate trend lines to model the variation of the thresholds for 5%, 10%, 15% and 20% FPRs at different window lengths.

Subsequently, to search for the threshold values for 5%, 10%, 15% and 20% FPRs in Approach 2, we iteratively reduced the threshold values starting at 1.5 with a step size of 0.001. During each iteration, we compared the thresholds against the predicted probabilities and deducted the product of the corresponding gradient of the trend line and the window number, from the current threshold value to account for changes in window lengths. This process continued until we found the threshold values that best aligned with the required FPRs. We started the threshold search at 1.5 to increase the threshold search space as the use of gradients would further decrease the thresholds.

### Implementation and evaluation of the MCNN on CTU-UHB

The MCNN proposed by Petrozziello et al.^14^ is the current state-of-the-art performing model to detect fetal compromise during labour. It was trained on the last 60 mins of over 30,000 CTG recordings of the Oxford dataset and validated its performance on the last 60 mins of the benchmark CTU-UHB dataset. Its architecture comprises two distinct 12-layer CNN branches designed to process FHR and UC signals separately. Additionally, it includes another branch featuring a single fully connected layer with an output size of 10, which accepts a quality vector of size 10 as the third input to the network. According to Petrozziello’s preliminary work^35^, the 12-layer CNN is composed of five convolutional layers with a ReLU activation function, interleaved with five max pooling layers to halve the size of each convolutional input. The final layer of each 12-layer CNN consists of a fully connected layer with an output size equivalent to the size of the quality vector. The quality values were computed over 15 mins moving windows of FHR with 5 mins increments across the 60 mins FHR. These quality values for each 15 mins window were determined as the ratio of valid signal points to the total FHR signal points in the raw 4Hz data. In the MCNN architecture, the output of the FHR CNN branch is multiplied by the output of the quality vector branch, and the resultant output is concatenated with the output of the UC CNN branch. This combined output then proceeds through two fully connected layers with an output size of 10, culminating in the final softmax layer, which provides class probabilities. While dropout and batch normalization layers are incorporated throughout the network, the exact locations of these layers within the network are not specified.

Our implementation of the MCNN as depicted in Fig. 6, closely followed the original MCNN architecture mentioned above. However, we opted for sigmoid as the final layer instead of softmax, as it is typically used for binary classification tasks. For each convolutional layer, we selected a kernel size of 9 and 40 filters based on the hyper-parameter optimization contour plot provided in the original MCNN implementation^14^. Batch normalization layers were inserted between each convolutional layer and its ReLU activation. Dropout layers were utilized between fully connected layers and before the last output layer of the MCNN, as well as before the last layer of each of the two branches of 12-layer CNNs to reduce the overfitting of the network. Although we did not incorporate UC in the FHR-LINet model, we utilized the last 60 mins of UC signals from the CTU-UHB dataset, downsampled at 0.25 Hz, in our implementation of MCNN to closely follow the original implementation of Petrozziello et al.^14^. We trained the MCNN using the same methodology applied to the FHR-LINet model, and the results were reported using five times repeated 5-fold cross-validation only on Approach 3, as its model architecture requires evaluating the same window length used during training.

### Evaluation of the performance of FHR-LINet and MCNN trained on different input lengths

The proposed FHR-LINet model is designed to be invariant to input length, allowing training with various FHR input lengths. To assess the performance of FHR-LINet when trained on different lengths of FHR, we trained three FHR-LINet models with different input lengths without data augmentation: the 60 mins of FHR, the first 45 mins and the first 30 mins of FHR. Subsequently, we independently assessed the performance of these three trained FHR-LINet models on the 60 mins test data using Approaches 1 and 2. However, in Approach 3, each trained model was assessed on test data of the same length as its trained data.

Additionally, we trained and assessed the MCNN on various lengths of CTU-UHB CTG recordings: the entire 60 mins, the first 45 mins (resulting in a TTP of 45 mins), and the first 30 mins (resulting in a TTP of 30 mins) using Approach 3. Since the MCNN is not input length invariant, we adjusted the model architecture based on the size of the quality vector obtained using 15 mins moving window segments with 5 mins increments to evaluate different lengths of CTG recordings. This involved training three distinct models. For instance, when evaluating the entire 60 mins of CTG, the output size of the three branches and two fully connected layers was set to 10, corresponding to the number of quality values calculated over the 60 mins duration. Consequently, for CTG lengths of 45 mins and 30 mins, the output size of the three branches and the two fully connected layers was adjusted to 7 and 4, respectively. We utilized five times repeated 5-fold cross-validation for training and reporting results.

### Implementation and evaluation of FHR feature based classification

In this study, we evaluate the performance of four FHR features using the three proposed Approaches. The four features we used are: DC_PRSA_ quantifying the downward trend of FHR, MAD_dtrd_ quantifying the average depth of decelerations, $\beta$_0_ representing the FHR baseline and H measuring FHR variability. Additionally, we evaluated the performance of all four features combined. The DC_PRSA_ was selected for our study as it is the primary feature used in the OxSys 1.5 algorithm^33^. To calculate the DC_PRSA_ values of FHR recordings, we used the parameter values used by Georgieva et al.^33^ in the phase rectified signal averaging method. The features, MAD_dtrd_, $\beta$_0_ and H of FHR were chosen for comparison as they were identified as the top-performing features for detecting fetal compromise compared to other features by Spilka et al.^17^. We utilized their original implementation, which is available online, to directly compute these features. The last 60 mins of the raw FHR recordings at 4Hz were used to calculate all four FHR features. Additionally, since Approach 1 and Approach 2 evaluate smaller window segments, the features were also calculated for each window segment evaluated in these approaches.

The features were trained using the Logistic Regression classifier as it generates class prediction probabilities on the testing data. This classifier was trained using the features computed from the last 60 mins of the raw FHR. Subsequently, these predicted class probabilities were employed with Approach 1, Approach 2, and Approach 3 to determine the final TPRs and TTP at 5%, 10%, 15%, and 20% FPRs. Training and reporting of results were conducted using five times repeated 5-fold cross-validation.

### Evaluation of the effect of signal loss using FHR-LINet model

The performance of the FHR-LINet model trained with augmented 30 mins data was evaluated using the three approaches at varying signal loss percentages in the FHR signal. The evaluation began at 10% signal loss and increased in increments of 10% up to 100%. To accomplish this, a separate model was trained for each signal loss threshold during the training stage. Any augmented 30 mins FHR segments in the training data that did not meet the signal loss threshold were removed from the training process.

During the evaluation stage, only model predictions of FHR window segments in the 60 mins test FHR recordings that met the corresponding signal loss threshold were accepted. If an FHR window segment did not meet the signal loss threshold, the prediction value of that window was set to zero. This means that FHR window segments failing to meet the signal loss thresholds were predicted as normal, regardless of the model’s prediction output for that window segment. The outcome prediction for the full FHR recordings was based on the individual predictions of window segments as specified in each of Approach 1, 2 and 3. All FHR segments in an FHR recording that met the signal loss threshold were given equal weighting during training and evaluation.

## Data Availability

Data is from the publicly available CTU-UHB dataset. It can be accessed at https://physionet.org/content/ctu-uhb-ctgdb/1.0.0/.
